# Supplementary material for: Drivers and Distribution of Henipavirus-Induced Syncytia: What Do We Know?
Source: Viruses. 2021 Sep 2;13(9):1755. doi: 10.3390/v13091755 (PMC8472861; doi:10.3390/v13091755)
Supplement: Supplementary file 1 [file viruses-13-01755-s001.zip › viruses-1303101-supplementary.pdf]

Supplementary Materials: Drivers and Distribution of Henipavirus-Induced Syncytia: What Do We Know?

Amandine Gamble [1,*], Yao Yu Yeo [2,†], Aubrey A. Butler [1,†], Hubert Tang [1], Celine E. Snedden [1], Christian T. Mason[3], David W. Buchholz [2], John Bingham [4], Hector C. Aguilar [2], and James O. Lloyd-Smith [1]

1. Meta-analysis methodology

We screened the Web of Science Core Collection database on October 29, 2020 using the following keywords: ("in vivo" OR infect* OR histo*) AND (henipavir* OR hendra OR nipah OR "equine morbilli*") (1 271 records). We also considered studies referenced in full-texts assessed for eligibility and potentially reporting datasets of interest (4 records). We then selected publications reporting data from in vivo experimental infections using HeV, NiV or CedV. To ensure that we included in our meta-analysis only studies that actively screened for syncytia, we excluded the publications that never mentioned syncytia, multinucleated cells or cell fusion in the method or result sections. Data collected from individuals who received a treatment (passive or active immunization or antiviral) or were inoculated deficient virus (e.g., knockout of non-structural proteins) were also excluded from the meta-analysis. The complete selection procedure is described in Figure S1 following the Preferred Reporting Items for Systematic Reviews and Meta-Analyses (PRISMA) [1]. Studies included in the quantitative analyses are listed in Table S1.

For each study, data on syncytium occurrence in tissue samples (assessed by histological analyses) were manually extracted from the publication text and tables. Data on virus culture, viral RNA detection (detected by reverse transcription polymerase chain reaction) or viral antigen detection (detected by immunohistochemstry) were also included in the data set when available. Data were compiled as detection/non-detection of syncytia and viral material in individual samples or as proportion of positive samples depending on the resolution at which data were reported. Syncytia were considered as detected if said so explicitly, and non-detected if reported that no syncytia were detected, no lesion were detected, or detected lesions were limited to X and Y (not including syncytia). When such explicit information was not available, syncytium data were recorded as not available (in particular, non-detection was not inferred from the absence of report of detection). Individual metadata were also included in the data set, in particular host species, viral strain, route and dose of inoculation and time between inoculation and euthanasia. Data availability is summarized in Figure S2.

Sample size was approximated by the minimum plausible sample size based on the available data if no exact sample size was available. Time post-infection was approximated by the rounded averaged value when the range of plausible value was of three days or less; if a range of more than three days was plausible, the data were excluded from any analysis or plot considering time post-infection. Syncytium prevalence was then calculated as the ratio of individuals in which syncytia were detected / individuals for which syncytia detection or non-detection is reported at least once for a given tissue, physiologic system, and/or time-point depending on the analysis.

We used the software R (version 3.6.2) [2] and packages `tydir` (version 1.1.2) [3], `ggplot2` (version 3.3.2) [4] and `cowplot` (version 1.0.0) [5] for data curation, analysis and visualization. The complete data set and the codes used to reproduce the figures and analyses are available at https://doi.org/10.5281/zenodo.5338802 [?].

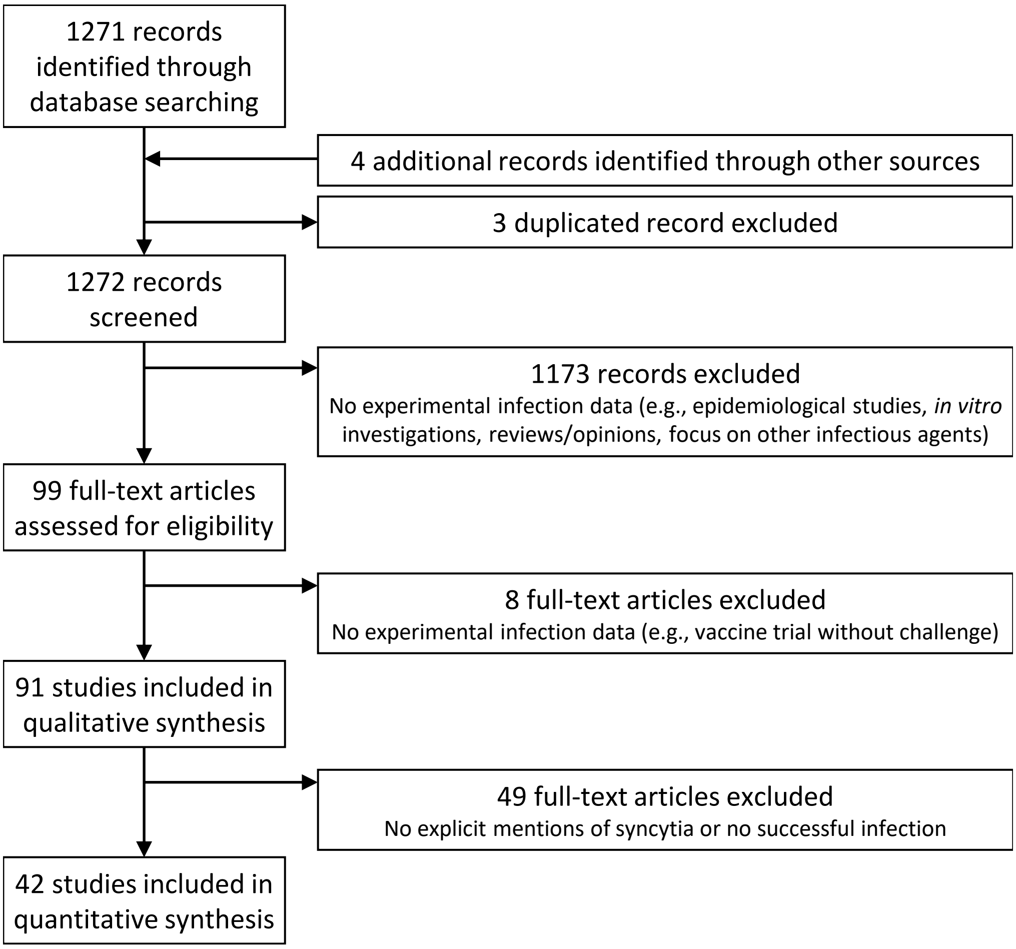

Figure S1. Selection process of the studies included in the meta-analysis of syncytium formation following experimental henipavirus infections in vivo.

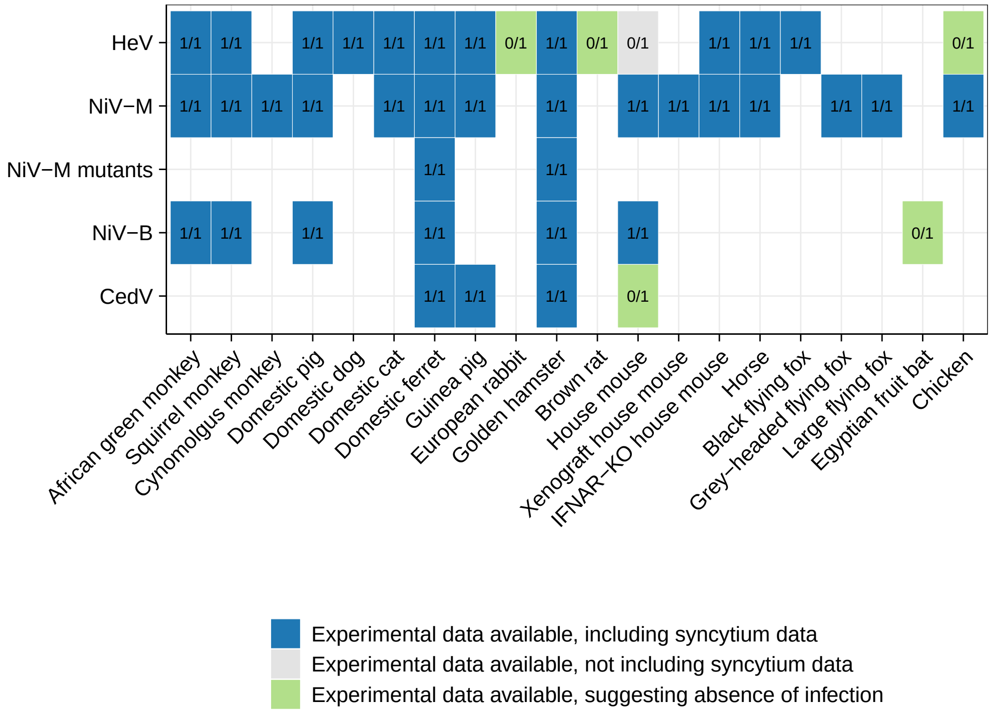

Figure S2. Map of syncytium data availability across host and virus species following experimental henipavirus infection in vivo. A publication was counted as reporting syncytium data if it either explicitly reported the observation or non-observation of syncytia, or if it reported the absence of any lesion from all the explored tissues. Numbers indicate the number of publications reported syncytium data / number of publications reporting experimental infection data. Blue cells indicate host-virus combinations for which at least one publications reported syncytium data; grey cells indicate combinations for which no syncytium data are available; and green cells indicate combinations for which experimental inoculation did not induce any host response (immune or pathological); blank spaces indicate that no data are available.

Table S1. 1/2. Studies included in the meta-analysis of syncytium formation following experimental henipavirus infections *in vivo*. $TCID_{50}$: median tissue-culture infectious dose; LD_{50}: median lethal dose; PFU: plaque forming units.

**Study**	**Species**	**Inoculation route**	Dose	**Virus**
Baseler et al. 2014 [6]	Hamster	Oronasal	1×10^7 $TCID_{50}$	NiV-M
Baseler et al. 2014 [6]	Hamster	Oronasal	1×10^7 $TCID_{50}$	NiV-B
Bossart et al. 2009 [7]	Ferret	Oronasal	500 $TCID_{50}$	NiV-M
Bossart et al. 2009 [7]	Ferret	Oronasal	5×10^4 $TCID_{50}$	NiV-M
Clayton et al. 2016 [8]	Ferret	Oronasal	5000 $TCID_{50}$	NiV-B
Clayton et al. 2016 [8]	Ferret	Oronasal	5000 $TCID_{50}$	NiV-M
Cong et al. 2017 [9]	African green monkey	Intratracheal	13000 PFU	NiV-M
Cong et al. 2017 [9]	African green monkey	Aerosol	40300 PFU	NiV-M
de Wit et al. 2014 [10]	Hamster	drinking	5×10^8 $TCID_{50}$	NiV-B
Escaffre et al. 2018 [11]	Hamster	Aerosol	85000 PFU	NiV-M
Escaffre et al. 2018 [11]	Hamster	Aerosol	2×10^4 PFU	NiV-M
Escaffre et al. 2018 [11]	Hamster	Aerosol	1×10^5 PFU	NiV-M
Geisbert et al. 2010 [12]	African green monkey	Intratracheal/oral	13×10^5 PFU	NiV-M
Geisbert et al. 2010 [12]	African green monkey	Intratracheal	23000 PFU	NiV-M
Geisbert et al. 2010 [12]	African green monkey	Intratracheal	2500 - 65000 PFU	NiV-M
Geisbert et al. 2010 [12]	African green monkey	Intratracheal/oral	700 - 13×10^5 PFU	NiV-M
Geisbert et al. 2010 [12]	African green monkey	Intratracheal	59000 PFU	NiV-M
Geisbert et al. 2010 [12]	African green monkey	Intratracheal/oral	81000 PFU	NiV-M
Geisbert et al. 2010 [12]	African green monkey	Intratracheal	65000 PFU	NiV-M
Geisbert et al. 2010 [12]	African green monkey	Intratracheal	2500 PFU	NiV-M
Geisbert et al. 2010 [12]	African green monkey	Intratracheal	7000 PFU	NiV-M
Geisbert et al. 2014 [13]	African green monkey	Intravenous	5×10^5 PFU	NiV-M
Geisbert et al. 2019 [14]	African green monkey	Intranasal	2×10^4 PFU	NiV-B
Geisbert et al. 2019 [14]	African green monkey	Intranasal	2000 PFU	NiV-B
Guillaume et al. 2009 [15]	Hamster	Intraperitoneal	1000 PFU	HeV
Guillaume-Vasselin et al. 2016 [16]	Hamster	Intraperitoneal	1×10^4 LD_{50}	HeV
Hammoud et al. 2018 [17]	African green monkey	Aerosol	100 PFU	NiV-M
Hammoud et al. 2018 [17]	African green monkey	Aerosol	1000 PFU	NiV-M
Hooper et al. 1997a [18]	Horse	Intranasal	Unknown	HeV
Hooper et al. 1997a [18]	Horse	Intravenous/aerosol	2×10^7 $TCID_{50}$	HeV
Hooper et al. 1997b [19]	Cat	Intranasal	5000 $TCID_{50}$	HeV
Hooper et al. 1997b [19]	Cat	Oral	5000 $TCID_{50}$	HeV
Hooper et al. 1997b [19]	Cat	Direct contact	5000 $TCID_{50}$	HeV
Hooper et al. 1997b [19]	Cat	Subcutaneous	5000 $TCID_{50}$	HeV
Hooper et al. 1997b [19]	Guinea pig	Subcutaneous	5000 $TCID_{50}$	HeV
Johnston et al. 2015 [20]	African green monkey	Intratracheal	25000 PFU	NiV-M
Li et al. 2010 [21]	Pig	Intranasal	2×10^7 PFU	HeV
Li et al. 2010 [21]	Pig	Oronasal	66×10^6 PFU	HeV

Table S1. 2/2. Studies included in the meta-analysis of syncytium formation following experimental henipavirus infections *in vivo*. $TCID_{50}$: median tissue-culture infectious dose; PFU: plaque forming units.

**Study**	**Species**	**Inoculation route**	Dose	**Virus**
McEachern et al. 2008 [22]	Cat	Oronasal	5×10^4 $TCID_{50}$	NiV-M
Middleton et al. 2007 [23]	Guinea pig	Intraperitoneal	5×10^4 $TCID_{50}$	NiV-M
Middleton et al. 2017 [24]	Dog	Oronasal	2×10^6 $TCID_{50}$	HeV
Mire et al. 2013 [25]	Ferret	Intranasal	5000 PFU	NiV-M
Mire et al. 2015 [26]	African green monkey	Intratracheal/intranasal	5×10^5 PFU	NiV-B
Mire et al. 2019 [27]	African green monkey	Intratracheal/intranasal	5×10^5 PFU	NiV-B
Mungall et al. 2006 [28]	Cat	Subcutaneous	5000 $TCID_{50}$	NiV-M
Mungall et al. 2006 [28]	Cat	Subcutaneous	500 $TCID_{50}$	NiV-M
Mungall et al. 2007 [29]	Cat	Subcutaneous	500 $TCID_{50}$	NiV-M
Munster et al. 2012 [30]	Hamster	Intranasal	1×10^5 $TCID_{50}$	NiV-M
Pallister et al. 2009 [31]	Ferret	Oronasal	5000 $TCID_{50}$	NiV-M
Prasad et al. 2019 [32]	African green monkey	Aerosol	1×10^4 PFU	NiV-B
Prasad et al. 2019 [32]	African green monkey	Aerosol	1000 PFU	NiV-B
Rockx et al. 2010 [33]	African green monkey	Intratracheal	4×10^5 $TCID_{50}$	HeV
Satterfield et al. 2015 [34]	Ferret	Intranasal	5000 PFU	NiV-M
Satterfield et al. 2016 [35]	Ferret	Intranasal	5000 PFU	NiV-M
Satterfield et al. 2019 [36]	Ferret	Intranasal	5000 PFU	NiV-M
Tanimura et al. 2006 [37]	Chicken	Allantoic	158489 PFU	NiV-M
Tanimura et al. 2006 [37]	Chicken	Yolk	7943 PFU	NiV-M
Torres-Velez et al. 2008 [38]	Guinea pig	Intraperitoneal	6×10^4 PFU	NiV-M
Valbuena et al. 2014 [39]	Xenograft mouse	Xenograft injection	Unknown	NiV-M
Weingartl et al. 2005 [40]	Pig	Intranasal/oral/ocular	25×10^4 PFU	NiV-M
Weingartl et al. 2006[41]	Pig	Intranasal	25×10^4 PFU	NiV-M
Westbury et al. 1995 [42]	Dog	Subcutaneous	5000 $TCID_{50}$	HeV
Westbury et al. 1995 [42]	rat	Subcutaneous	5000 $TCID_{50}$	HeV
Westbury et al. 1995 [42]	rabbit	Subcutaneous	5000 $TCID_{50}$	HeV
Westbury et al. 1995 [42]	house_mouse	Subcutaneous	5000 $TCID_{50}$	HeV
Westbury et al. 1995 [42]	Chicken	Subcutaneous	5000 $TCID_{50}$	HeV
Westbury et al. 1996 [43]	Cat	Oral/intranasal/subcutaneous	3981 $TCID_{50}$	HeV
Williamson et al. 2000 [44]	Guinea pig	Subcutaneous	5×10^4 $TCID_{50}$	HeV
Williamson et al. 2001 [45]	Guinea pig	Subcutaneous	5×10^4 $TCID_{50}$	HeV
Williamson et al. 2001 [45]	Guinea pig	Subcutaneous	3×10^4 $TCID_{50}$	HeV
Wong et al. 2003 [46]	Hamster	Intraperitoneal	270 PFU	NiV-M
Wong et al. 2003 [46]	Hamster	Unknown	Unknown	NiV-M
Woon et al. 2020 [47]	Ferret	Oral/intranasal	3×10^4 $TCID_{50}$	HeV
Woon et al. 2020 [47]	Black flying fox	Oral/intranasal	3×10^4 $TCID_{50}$	HeV
Yoneda et al. 2013 [48]	African green monkey	Intraperitoneal	1×10^6 $TCID_{50}$	NiV-M

2. Additional Results

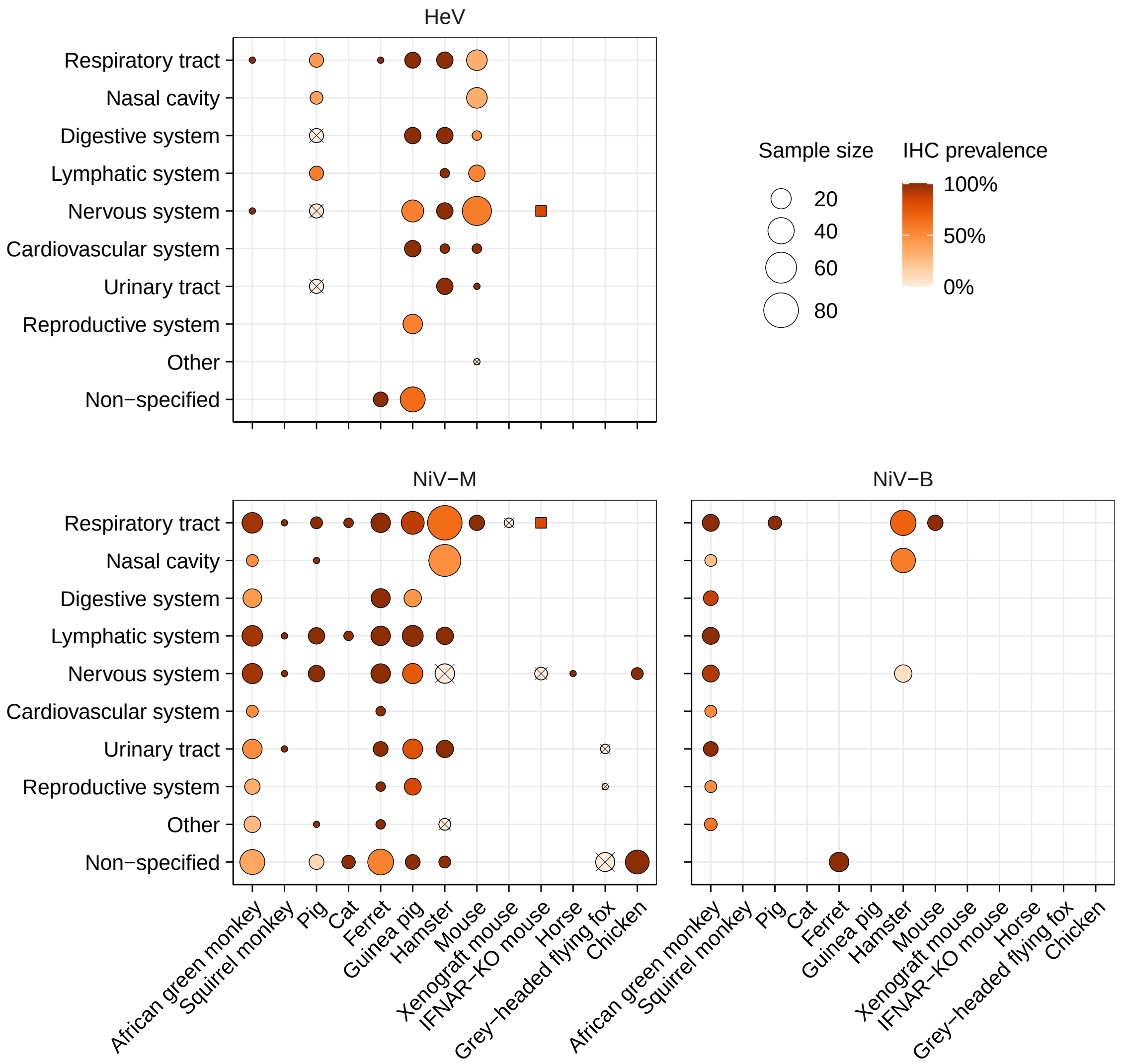

Figure S3. Proportion of individuals presenting viral antigens detected by immunohistochemistry (IHC) following experimental henipavirus infection in vivo. Dark orange squares indicate that viral antigens were detected but the exact proportion of individuals is unknown; blank spaces indicate that no data are available. Data obtained using different inoculation routes or doses and collected at different time post-infection were merged together. This figure represents a realistic map of potential syncytium data, as syncytia are detected by histology, which uses similar sample collection and preparation procedures that IHC (minus the staining of viral proteins via immunolabeling). Note that the scale for sample size is different from the figures reporting syncytium data due to larger sample sizes available for IHC. Data were execrated from the studies listed in S1.

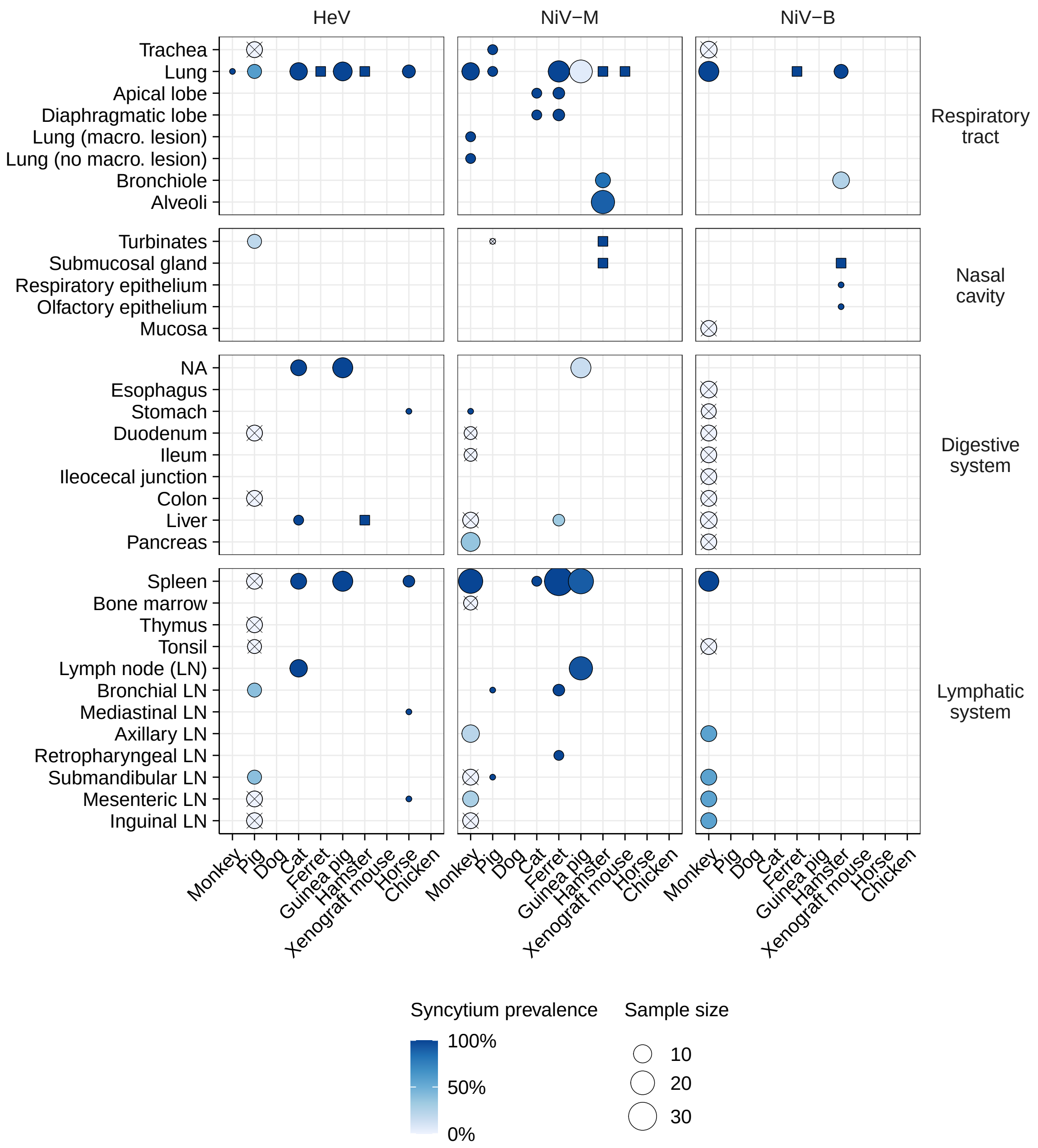

Figure S4. 1/2. Proportion of individuals presenting syncytia in different tissues following experimental henipavirus infection in vivo. Tissues are broken down at with varying levels of resolution (e.g., brain versus hippocampus, despite the hippocampus being part of the brain) corresponding to the diversity of resolution levels used across studies. Dot size indicates the number of individuals for which information regarding the detection or non-detection of syncytia was available, and dot color indicates the proportion of individuals presenting syncytia among those. Dark blue squares indicate that syncytia have been reported but that the exact proportion of individuals was unknown; blank spaces indicate that no data are available. Macro.: macroscopic; GI tract: gastro-intestinal tract. Data were execrated from the studies listed in S1.

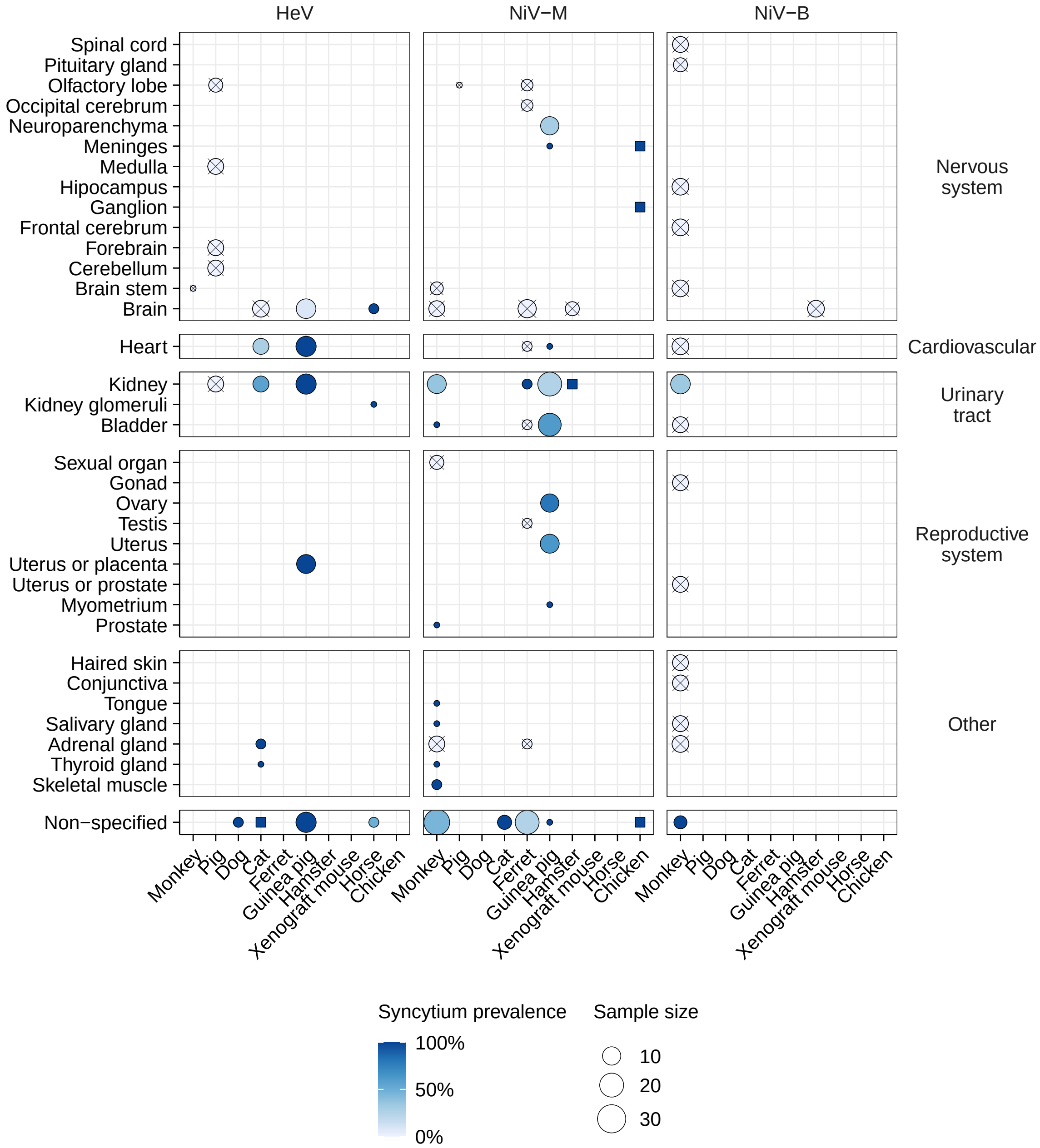

Figure S4. 2/2. Proportion of individuals presenting syncytia in different tissues following experimental henipavirus infection in vivo. Tissues are broken down at with varying levels of resolution (e.g., brain versus hippocampus, despite the hippocampus being part of the brain) corresponding to the diversity of resolution levels used across studies. Dot size indicates the number of individuals for which information regarding the detection or non-detection of syncytia was available, and dot color indicates the proportion of individuals presenting syncytia among those. Dark blue squares indicate that syncytia have been reported but that the exact proportion of individuals was unknown; blank spaces indicate that no data are available. Macro.: macroscopic.

The proportion of individuals presenting syncytia over time post-infection broken down by study is represented in Figures S5–S7. The proportion of individuals presenting syncytia was calculated by dividing the number of individuals for which syncytia were detected by the number of individuals for which information regarding the detection or non-detection of syncytia was available, for each given tissue and at each given time-point. If several samples were collected from a given individual, the individual was considered as presenting syncytia if syncytia were detected in at least one sample (e.g., if several lymph nodes were collected from an individual and syncytia were detected in at least one, this individual was considered as presenting syncytia in lymphatic tissues). "Cardiovascular system" corresponds to heart and major blood vessels. "Other" regroups conjunctiva, skin, lip, pharynx, salivary gland and thyroid samples. "Organ mix" corresponds to unspecified tissues.

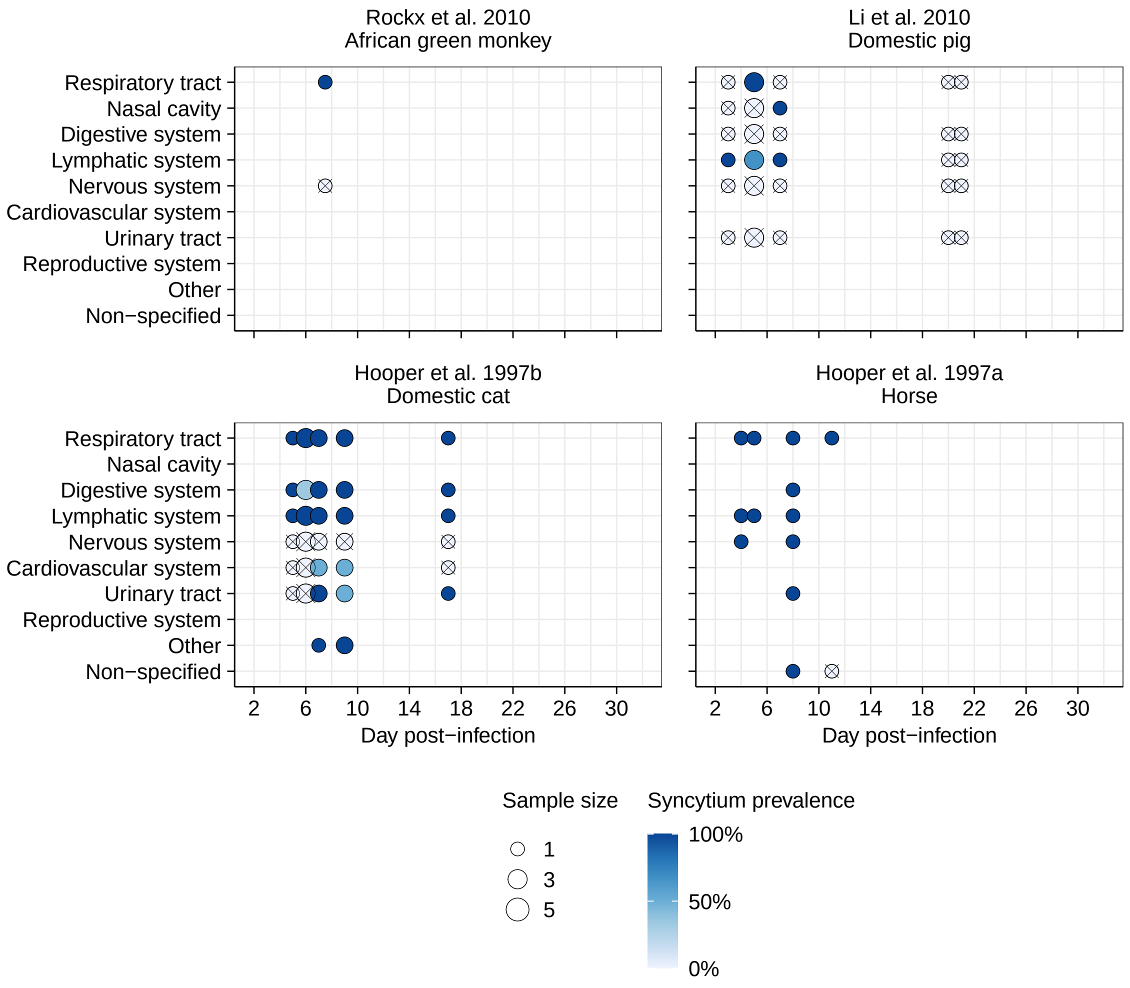

Figure S5. 1/2. Proportion of individuals presenting syncytia over time post-infection following experimental Hendra virus infection in vivo. Dot size indicates the number of individuals for which information regarding the detection or non-detection of syncytia was available, and dot color indicates the proportion of individuals presenting syncytia among those. Crossed circles indicate that no syncytia were observed; dark blue squares indicate that syncytia have been reported but that the exact proportion of individuals was unknown; blank spaces indicate that no data are available.

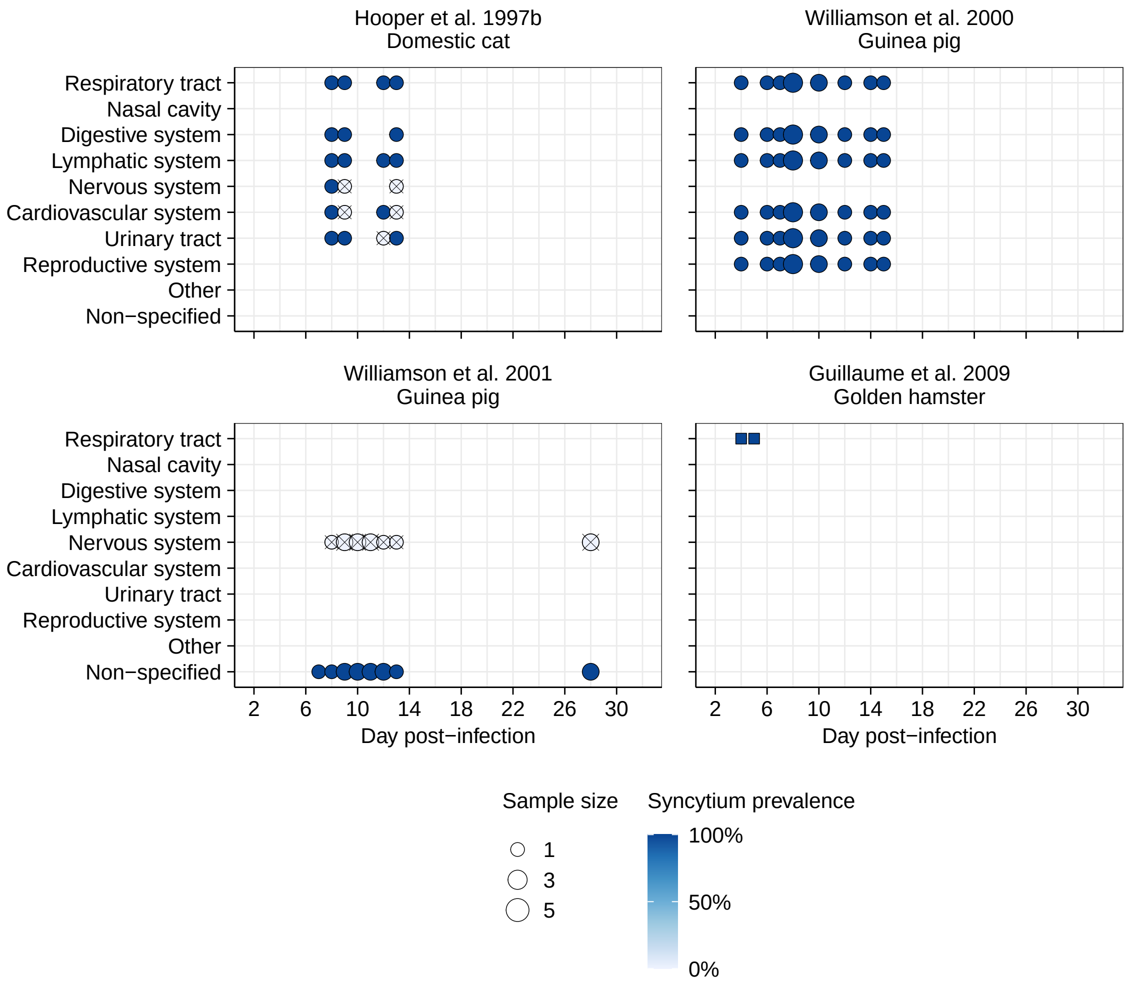

Figure S5. 2/2. Proportion of individuals presenting syncytia over time post-infection following experimental Hendra virus infection in vivo. Dot size indicates the number of individuals for which information regarding the detection or non-detection of syncytia was available, and dot color indicates the proportion of individuals presenting syncytia among those. Crossed circles indicate that no syncytia were observed; dark blue squares indicate that syncytia have been reported but that the exact proportion of individuals was unknown; blank spaces indicate that no data are available.

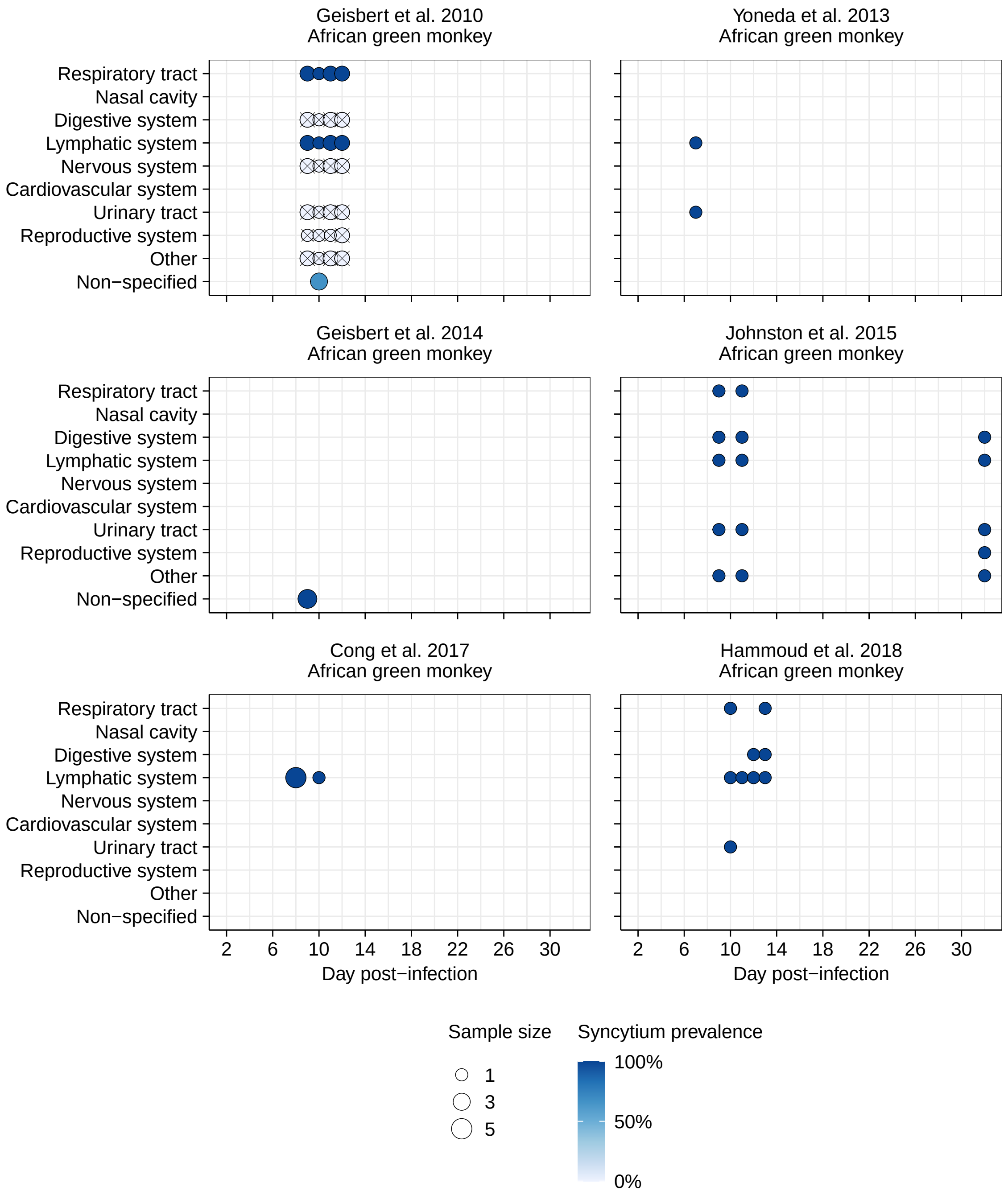

Figure S6. 1/3. Proportion of individuals presenting syncytia over time post-infection following experimental Nipah Malaysia virus infection in vivo. Dot size indicates the number of individuals for which information regarding the detection or non-detection of syncytia was available, and dot color indicates the proportion of individuals presenting syncytia among those. Crossed circles indicate that no syncytia were observed; dark blue squares indicate that syncytia have been reported but that the exact proportion of individuals was unknown; blank spaces indicate that no data are available.

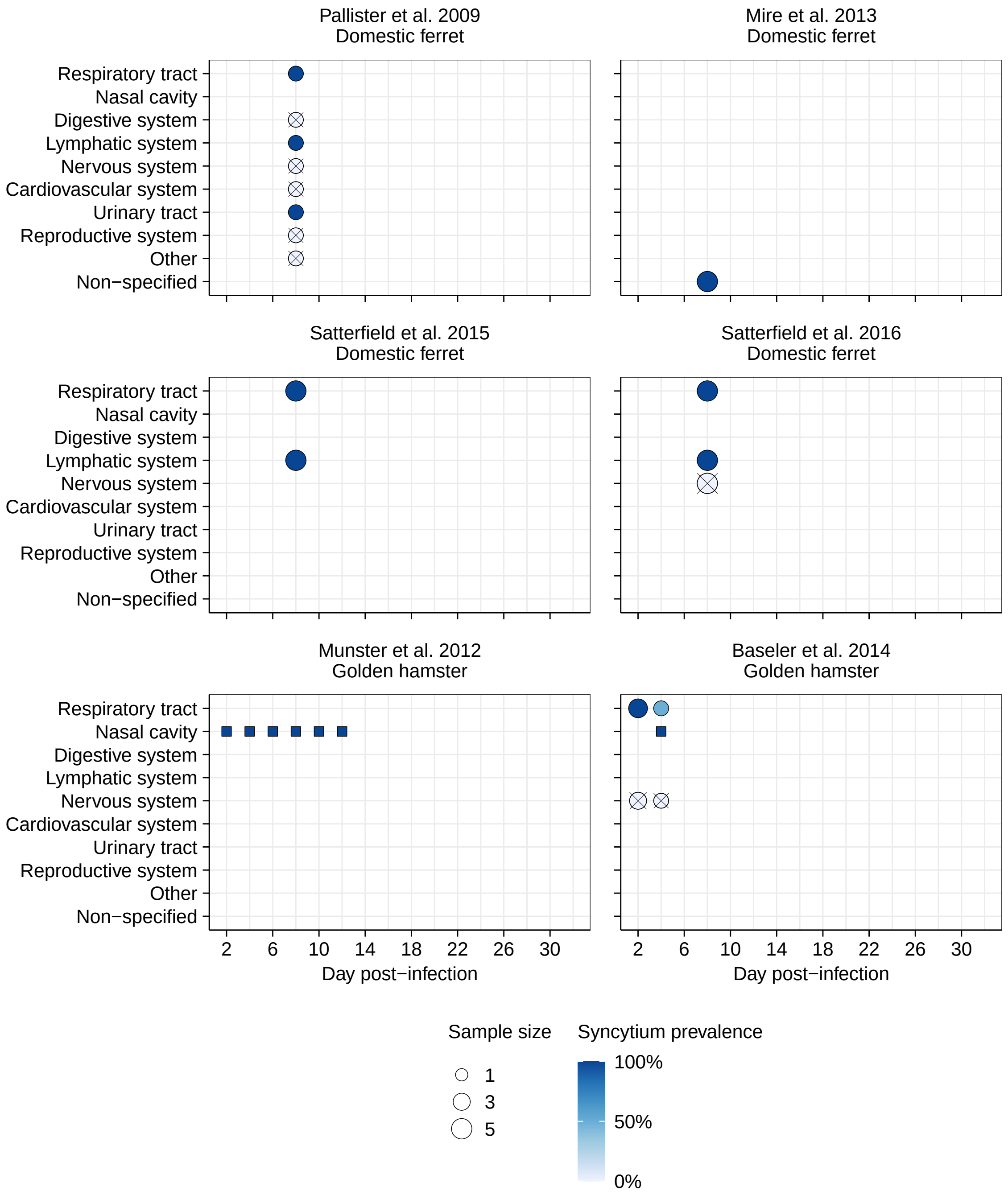

Figure S6. 2/3. Proportion of individuals presenting syncytia over time post-infection following experimental Nipah Malaysia virus infection in vivo. Dot size indicates the number of individuals for which information regarding the detection or non-detection of syncytia was available, and dot color indicates the proportion of individuals presenting syncytia among those. Crossed circles indicate that no syncytia were observed; dark blue squares indicate that syncytia have been reported but that the exact proportion of individuals was unknown; blank spaces indicate that no data are available.

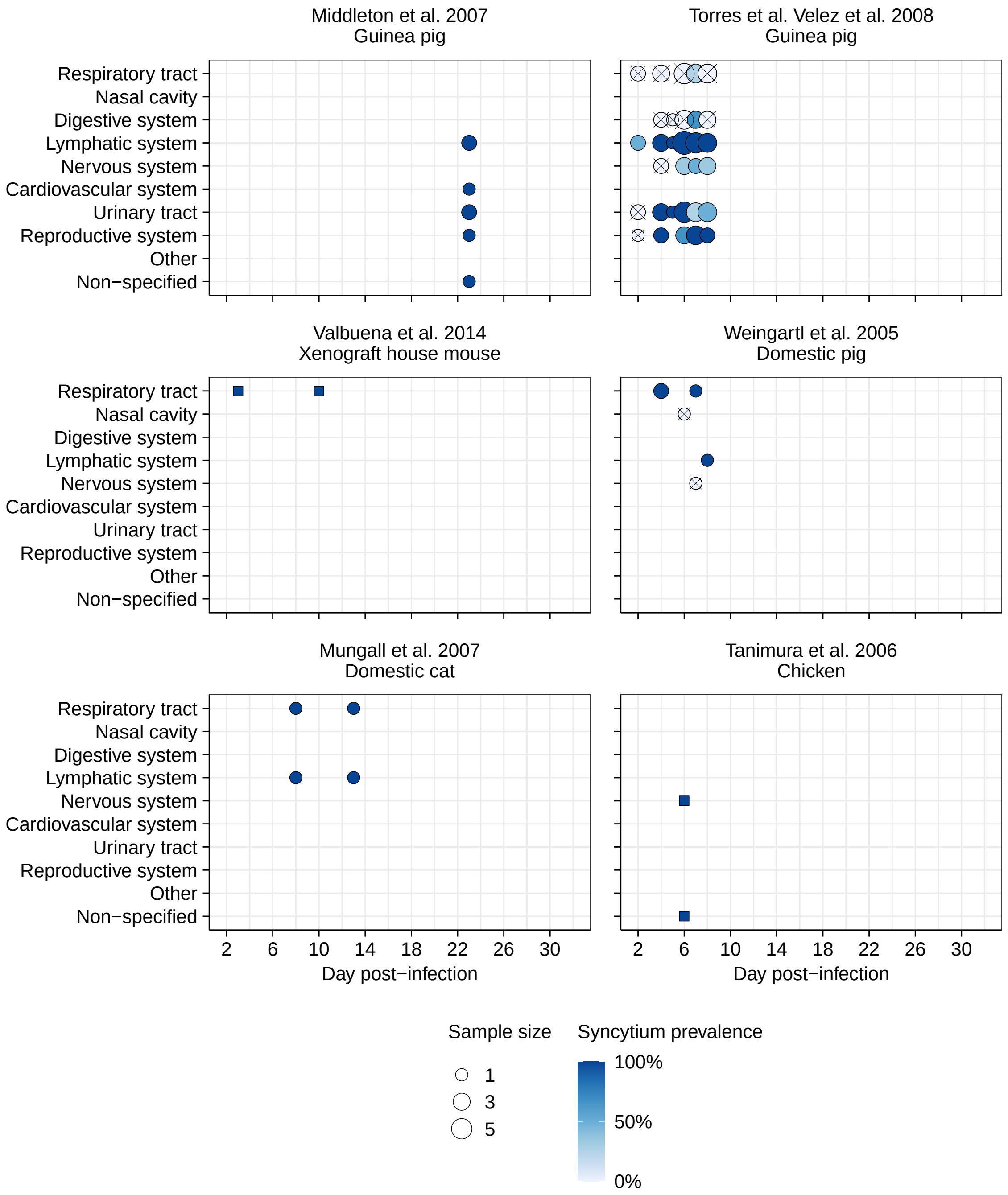

Figure S6. 3/3. Proportion of individuals presenting syncytia over time post-infection following experimental Nipah Malaysia virus infection in vivo. Dot size indicates the number of individuals for which information regarding the detection or non-detection of syncytia was available, and dot color indicates the proportion of individuals presenting syncytia among those. Crossed circles indicate that no syncytia were observed; dark blue squares indicate that syncytia have been reported but that the exact proportion of individuals was unknown; blank spaces indicate that no data are available.

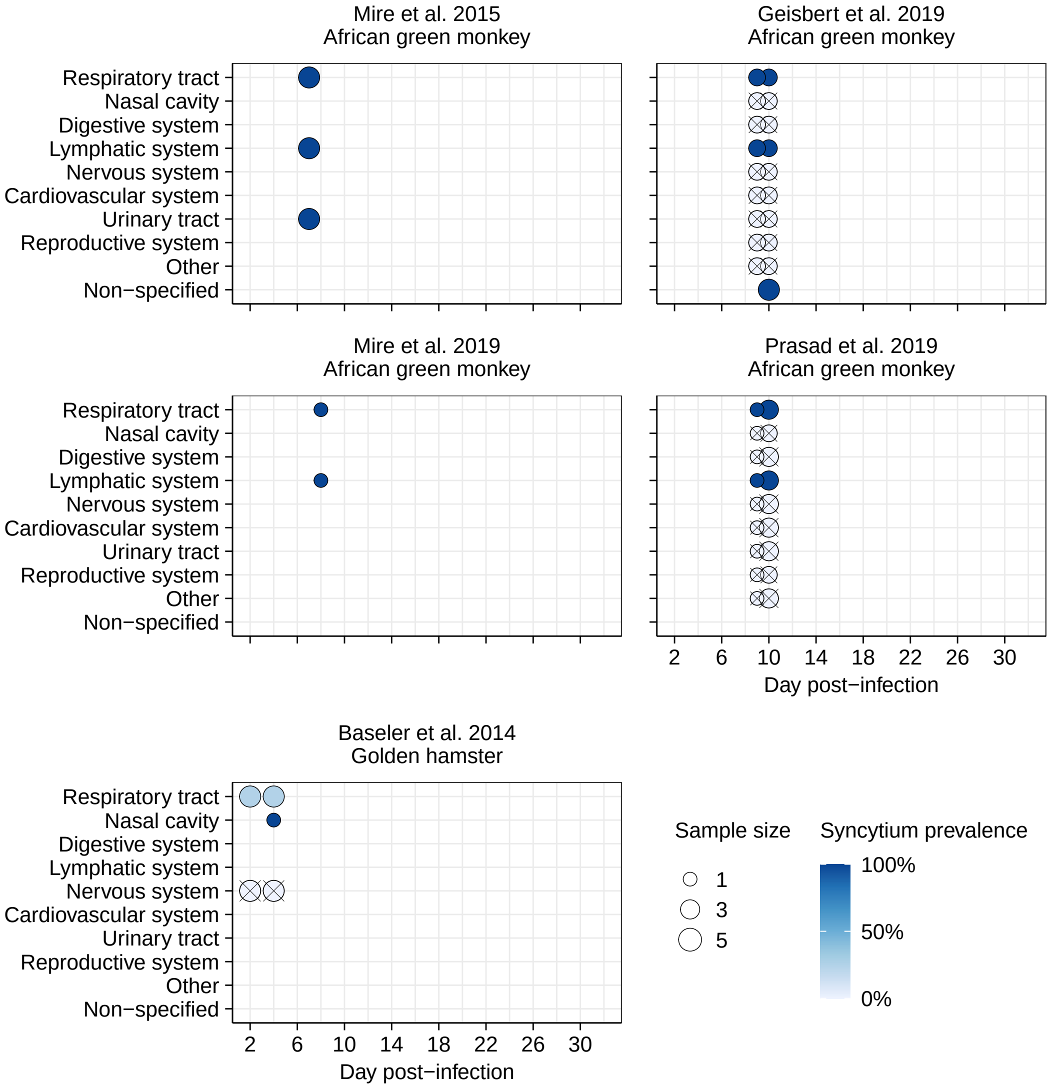

Figure S7. Proportion of individuals presenting syncytia over time post-infection following experimental Nipah Bangladesh virus infection in vivo. Dot size indicates the number of individuals for which information regarding the detection or non-detection of syncytia was available, and dot color indicates the proportion of individuals presenting syncytia among those. Crossed circles indicate that no syncytia were observed; dark blue squares indicate that syncytia have been reported but that the exact proportion of individuals was unknown; blank spaces indicate that no data are available.

1. Moher, D.; Liberati, A.; Tetzlaff, J.; Altman, D.G. Preferred Reporting Items for Systematic Reviews and Meta-Analyses: The PRISMA Statement. *PLoS Medicine* **2009**, *6*, 6.
2. R Development Core Team. *R: A Language and Environment for Statistical Computing*. R Foundation for Statistical Computing, Vienna, Austria, 2008. ISBN 3-900051-07-0.
3. Wickham, H. Tidyr: tidy messy data. R package version 1.1.2, 2020.
4. Wickham, H. *ggplot2: Elegant Graphics for Data Analysis*; Springer-Verlag New York, 2016.
5. Wilke, C.O. cowplot: Streamlined Plot Theme and Plot Annotations for "ggplot2". R package version 0.9. 4, 2019.
6. Baseler, L.; de Wit, E.; Scott, D.P.; Munster, V.J.; Feldmann, H. Syrian hamsters (Mesocricetus auratus) oronasally inoculated with a Nipah virus isolate from Bangladesh or Malaysia develop similar respiratory tract lesions. *Veterinary pathology* **2015**, *52*, 38–45.
7. Bossart, K.N.; Zhu, Z.; Middleton, D.; Klippel, J.; Crameri, G.; Bingham, J.; McEachern, J.A.; Green, D.; Hancock, T.J.; Chan, Y.P.; Hickey, A.C.; Dimitrov, D.S.; Wang, L.F.; Broder, C.C. A Neutralizing Human Monoclonal Antibody Protects against Lethal Disease in a New Ferret Model of Acute Nipah Virus Infection. *PLOS Pathogens* **2009**, p. e1000642. doi:10.1371/journal.ppat.1000642.
8. Clayton, B.A.; Middleton, D.; Arkinstall, R.; Frazer, L.; Wang, L.F.; Marsh, G.A. The nature of exposure drives transmission of Nipah viruses from Malaysia and Bangladesh in ferrets. *PLoS neglected tropical diseases* **2016**, *10*, e0004775.
9. Cong, Y.; Lentz, M.R.; Lara, A.; Alexander, I.; Bartos, C.; Bohannon, J.K.; Hammoud, D.; Huzella, L.; Jahrling, P.B.; Janosko, K.; Jett, C.; Kollins, E.; Lackemeyer, M.; Mollura, D.; Ragland, D.; Rojas, O.; Solomon, J.; Xu, Z.; Munster, V.; Holbrook, M.R. Loss in Lung Volume and Changes in the Immune Response Demonstrate Disease Progression in African Green Monkeys Infected by Small-Particle Aerosol and Intratracheal Exposure to Nipah Virus. *PLOS Neglected Tropical Diseases* **2017**, *11*, e0005532. doi:10.1371/journal.pntd.0005532.
10. de Wit, E.; Prescott, J.; Falzarano, D.; Bushmaker, T.; Scott, D.; Feldmann, H.; Munster, V.J. Foodborne Transmission of Nipah Virus in Syrian Hamsters. *PLoS pathogens* **2014**, *10*, e1004001. doi:10.1371/journal.ppat.1004001.
11. Escaffre, O.; Hill, T.; Ikegami, T.; Juelich, T.L.; Smith, J.K.; Zhang, L.; Perez, D.E.; Atkins, C.; Park, A.; Lawrence, W.S.; Sivasubramani, S.K.; Peel, J.E.; Peterson, J.W.; Lee, B.; Freiberg, A.N. Experimental Infection of Syrian Hamsters With Aerosolized Nipah Virus. *The Journal of Infectious Diseases* **2018**, *218*, 1602–1610. doi:10.1093/infdis/jiy357.
12. Geisbert, T.W.; Daddario-DiCaprio, K.M.; Hickey, A.C.; Smith, M.A.; Chan, Y.P.; Wang, L.F.; Mattapallil, J.J.; Geisbert, J.B.; Bossart, K.N.; Broder, C.C. Development of an Acute and Highly Pathogenic Nonhuman Primate Model of Nipah Virus Infection. *PLoS ONE* **2010**, *5*, e10690. doi:10.1371/journal.pone.0010690.
13. Geisbert, T.W.; Mire, C.E.; Geisbert, J.B.; Chan, Y.P.; Agans, K.N.; Feldmann, F.; Fenton, K.A.; Zhu, Z.; Dimitrov, D.S.; Scott, D.P.; Bossart, K.N.; Feldmann, H.; Broder, C.C. Therapeutic Treatment of Nipah Virus Infection in Nonhuman Primates with a Neutralizing Human Monoclonal Antibody. *Science Translational Medicine* **2014**, *6*, 242ra82–242ra82. doi:10.1126/scitranslmed.3008929.
14. Geisbert, J.B.; Borisevich, V.; Prasad, A.N.; Agans, K.N.; Foster, S.L.; Deer, D.J.; Cross, R.W.; Mire, C.E.; Geisbert, T.W.; Fenton, K.A. An Intranasal Exposure Model of Lethal Nipah Virus Infection in African Green Monkeys. *The Journal of Infectious Diseases* **2019**, p. jiz391. doi:10.1093/infdis/jiz391.
15. Guillaume, V.; Wong, K.T.; Looi, R.; Georges-Courbot, M.C.; Barrot, L.; Buckland, R.; Wild, T.F.; Horvat, B. Acute Hendra virus infection: Analysis of the pathogenesis and passive antibody protection in the hamster model. *Virology* **2009**, *387*, 459–465.
16. Guillaume-Vasselin, V.; Lemaitre, L.; Dhondt, K.P.; Tedeschi, L.; Poulard, A.; Charreyre, C.; Horvat, B. Protection from Hendra virus infection with Canarypox recombinant vaccine. *npj Vaccines* **2016**, *1*, 1–8.
17. Hammoud, D.A.; Lentz, M.R.; Lara, A.; Bohannon, J.K.; Feuerstein, I.; Huzella, L.; Jahrling, P.B.; Lackemeyer, M.; Laux, J.; Rojas, O.; Sayre, P.; Solomon, J.; Cong, Y.; Munster, V.; Holbrook, M.R. Aerosol Exposure to Intermediate Size Nipah Virus Particles Induces Neurological Disease in African Green Monkeys. *PLOS Neglected Tropical Diseases* **2018**, *12*, e0006978. doi:10.1371/journal.pntd.0006978.
18. Hooper, P.; Ketterer, P.; Hyatt, A.; Russell, G. Lesions of experimental equine morbillivirus pneumonia in horses. *Veterinary Pathology* **1997**, *34*, 312–322.
19. Hooper, P.; Westbury, H.; Russell, G. The lesions of experimental equine morbillivirus disease in cats and guinea pigs. *Veterinary pathology* **1997**, *34*, 323–329.
20. Johnston, S.C.; Briese, T.; Bell, T.M.; Pratt, W.D.; Shamblin, J.D.; Esham, H.L.; Donnelly, G.C.; Johnson, J.C.; Hensley, L.E.; Lipkin, W.I.; Honko, A.N. Detailed Analysis of the African Green Monkey Model of Nipah Virus Disease. *PLOS ONE* **2015**, *10*, e0117817. doi:10.1371/journal.pone.0117817.
21. Li, M.; Embury-Hyatt, C.; Weingartl, H.M. Experimental inoculation study indicates swine as a potential host for Hendra virus. *Veterinary research* **2010**, *41*, 33.
22. McEachern, J.A.; Bingham, J.; Crameri, G.; Green, D.J.; Hancock, T.J.; Middleton, D.; Feng, Y.R.; Broder, C.C.; Wang, L.F.; Bossart, K.N. A Recombinant Subunit Vaccine Formulation Protects against Lethal Nipah Virus Challenge in Cats. *Vaccine* **2008**, *26*, 3842–3852. doi:10.1016/j.vaccine.2008.05.016.
23. Middleton, D.; Morrissy, C.; van der Heide, B.; Russell, G.; Braun, M.; Westbury, H.; Halpin, K.; Daniels, P. Experimental Nipah Virus Infection in Pteropid Bats (Pteropus Poliocephalus). *Journal of Comparative Pathology* **2007**, *136*, 266–272. doi:10.1016/j.jcpa.2007.03.002.

24. Middleton, D.; Riddell, S.; Klein, R.; Arkinstall, R.; Haining, J.; Frazer, L.; Mottley, C.; Evans, R.; Johnson, D.; Pallister, J. Experimental Hendra Virus Infection of Dogs: Virus Replication, Shedding and Potential for Transmission. *Australian Veterinary Journal* **2017**, *95*, 10–18. doi:10.1111/avj.12552.
25. Mire, C.E.; Versteeg, K.M.; Cross, R.W.; Agans, K.N.; Fenton, K.A.; Whitt, M.A.; Geisbert, T.W. Single Injection Recombinant Vesicular Stomatitis Virus Vaccines Protect Ferrets against Lethal Nipah Virus Disease. *Virology Journal* **2013**, *10*, 353. doi:10.1186/1743-422X-10-353.
26. Mire, C.E.; Satterfield, B.A.; Geisbert, J.B.; Agans, K.N.; Borisevich, V.; Yan, L.; Chan, Y.P.; Cross, R.W.; Fenton, K.A.; Broder, C.C.; Geisbert, T.W. Pathogenic Differences between Nipah Virus Bangladesh and Malaysia Strains in Primates: Implications for Antibody Therapy. *Scientific Reports* **2016**, *6*. doi:10.1038/srep30916.
27. Mire, C.E.; Geisbert, J.B.; Agans, K.N.; Versteeg, K.M.; Deer, D.J.; Satterfield, B.A.; Fenton, K.A.; Geisbert, T.W. Use of single-injection recombinant vesicular stomatitis virus vaccine to protect nonhuman primates against lethal Nipah virus disease. *Emerging infectious diseases* **2019**, *25*, 1144.
28. Mungall, B.A.; Middleton, D.; Crameri, G.; Bingham, J.; Halpin, K.; Russell, G.; Green, D.; McEachern, J.; Pritchard, L.I.; Eaton, B.T.; Wang, L.F.; Bossart, K.N.; Broder, C.C. Feline Model of Acute Nipah Virus Infection and Protection with a Soluble Glycoprotein-Based Subunit Vaccine. *Journal of Virology* **2006**, *80*, 12293–12302. doi:10.1128/JVI.01619-06.
29. Mungall, B.A.; Middleton, D.; Crameri, G.; Halpin, K.; Bingham, J.; Eaton, B.T.; Broder, C.C. Vertical Transmission and Fetal Replication of Nipah Virus in an Experimentally Infected Cat. *The Journal of Infectious Diseases* **2007**, *196*, 812–816. doi:10.1086/520818.
30. Munster, V.J.; Prescott, J.B.; Bushmaker, T.; Long, D.; Rosenke, R.; Thomas, T.; Scott, D.; Fischer, E.R.; Feldmann, H.; De Wit, E. Rapid Nipah virus entry into the central nervous system of hamsters via the olfactory route. *Scientific reports* **2012**, *2*, 736.
31. Pallister, J.; Middleton, D.; Crameri, G.; Yamada, M.; Klein, R.; Hancock, T.J.; Foord, A.; Shiell, B.; Michalski, W.; Broder, C.C.; Wang, L.F. Chloroquine Administration Does Not Prevent Nipah Virus Infection and Disease in Ferrets. *Journal of Virology* **2009**, *83*, 11979–11982. doi:10.1128/JVI.01847-09.
32. Prasad, A.N.; Agans, K.N.; Sivasubramani, S.K.; Geisbert, J.B.; Borisevich, V.; Mire, C.E.; Lawrence, W.S.; Fenton, K.A.; Geisbert, T.W. A Lethal Aerosol Exposure Model of Nipah Virus Strain Bangladesh in African Green Monkeys. *The Journal of Infectious Diseases* **2019**, p. jiz469. doi:10.1093/infdis/jiz469.
33. Rockx, B.; Bossart, K.N.; Feldmann, F.; Geisbert, J.B.; Hickey, A.C.; Brining, D.; Callison, J.; Safronetz, D.; Marzi, A.; Kercher, L.; Long, D.; Broder, C.C.; Feldmann, H.; Geisbert, T.W. A Novel Model of Lethal Hendra Virus Infection in African Green Monkeys and the Effectiveness of Ribavirin Treatment. *Journal of Virology* **2010**, *84*, 9831–9839. doi:10.1128/JVI.01163-10.
34. Satterfield, B.A.; Cross, R.W.; Fenton, K.A.; Agans, K.N.; Basler, C.F.; Geisbert, T.W.; Mire, C.E. The Immunomodulating V and W Proteins of Nipah Virus Determine Disease Course. *Nature Communications* **2015**, *6*, 7483. doi:10.1038/ncomms8483.
35. Satterfield, B.A.; Cross, R.W.; Fenton, K.A.; Borisevich, V.; Agans, K.N.; Deer, D.J.; Graber, J.; Basler, C.F.; Geisbert, T.W.; Mire, C.E. Nipah Virus C and W Proteins Contribute to Respiratory Disease in Ferrets. *Journal of Virology* **2016**, *90*, 6326–6343. doi:10.1128/JVI.00215-16.
36. Satterfield, B.A.; Borisevich, V.; Foster, S.L.; Rodriguez, S.E.; Cross, R.W.; Fenton, K.A.; Agans, K.N.; Basler, C.F.; Geisbert, T.W.; Mire, C.E. Antagonism of STAT1 by Nipah virus P gene products modulates disease course but not lethal outcome in the ferret model. *Scientific reports* **2019**, *9*, 1–18.
37. Tanimura, N.; Imada, T.; Kashiwazaki, Y.; Sharifah, S. Distribution of viral antigens and development of lesions in chicken embryos inoculated with Nipah virus. *Journal of comparative pathology* **2006**, *135*, 74–82.
38. Torres-Velez, F.J.; Shieh, W.J.; Rollin, P.E.; Morken, T.; Brown, C.; Ksiazek, T.G.; Zaki, S.R. Histopathologic and Immunohistochemical Characterization of Nipah Virus Infection in the Guinea Pig. *Veterinary Pathology* **2008**, *45*, 576–585. doi:10.1354/vp.45-4-576.
39. Valbuena, G.; Halliday, H.; Borisevich, V.; Goez, Y.; Rockx, B. A human lung xenograft mouse model of Nipah virus infection. *PLoS pathogens* **2014**, *10*, e1004063.
40. Weingartl, H.; Czub, S.; Copps, J.; Berhane, Y.; Middleton, D.; Marszal, P.; Gren, J.; Smith, G.; Ganske, S.; Manning, L.; Czub, M. Invasion of the Central Nervous System in a Porcine Host by Nipah Virus. *Journal of Virology* **2005**, *79*, 7528–7534. doi:10.1128/JVI.79.12.7528-7534.2005.
41. Weingartl, H.M.; Berhane, Y.; Caswell, J.L.; Loosmore, S.; Audonnet, J.C.; Roth, J.A.; Czub, M. Recombinant Nipah Virus Vaccines Protect Pigs against Challenge. *Journal of Virology* **2006**, *80*, 7929–7938. doi:10.1128/JVI.00263-06.
42. Westbury, H.; Hooper, P.T.; Selleck, P.; Murray, P. Equine morbillivirus pneumonia: susceptibility of laboratory animals to the virus. *Australian Veterinary Journal* **1995**, *72*, 278–279.
43. Westbury, H.; Hooper, P.; Brouwer, S.; Selleck, P. Susceptibility of cats to equine morbillivirus. *Australian veterinary journal* **1996**, *74*, 132–134.
44. Williamson, M.; Hooper, P.; Selleck, P.; Westbury, H.; Slocombe, R. Experimental hendra virus infectionin pregnant guinea-pigs and fruit Bats (Pteropus poliocephalus). *Journal of comparative pathology* **2000**, *122*, 201–207.
45. Williamson, M.; Hooper, P.; Selleck, P.; Westbury, H.; Slocombe, R. A Guinea-Pig Model of Hendra Virus Encephalitis. *Journal of Comparative Pathology* **2001**, *124*, 273–279. doi:10.1053/jcpa.2001.0464.
46. Wong, K.T.; Grosjean, I.; Brisson, C.; Blanquier, B.; Fevre-Montange, M.; Bernard, A.; Loth, P.; Georges-Courbot, M.C.; Chevallier, M.; Akaoka, H.; others. A golden hamster model for human acute Nipah virus infection. *The American journal of pathology* **2003**, *163*, 2127–2137.

47. Woon, A.P.; Boyd, V.; Todd, S.; Smith, I.; Klein, R.; Woodhouse, I.B.; Riddell, S.; Crameri, G.; Bingham, J.; Wang, L.F.; others. Acute experimental infection of bats and ferrets with Hendra virus: Insights into the early host response of the reservoir host and susceptible model species. *PLoS Pathogens* **2020**, *16*, e1008412.
48. Yoneda, M.; Guillaume, V.; Ikeda, F.; Sakuma, Y.; Sato, H.; Wild, T.F.; Kai, C. Establishment of a Nipah virus rescue system. *Proceedings of the National Academy of Sciences* **2006**, *103*, 16508–16513.
